# Dorsomedial Striatal Scube1 Expression Correlates With Perseveration in a Rat Reversal-Learning Task

**DOI:** 10.7759/cureus.97327

**Published:** 2025-11-20

**Authors:** Abdullah Shakeel, Bekim Arifaj, Oluwaseun Akinniranye, Elektra Tsivitanidou, Mohammed B Khan, Olusegun Akinniranye

**Affiliations:** 1 Psychology, University of Cambridge, Cambridge, GBR; 2 Surgery, Princess Alexandra Hospital, Harlow, GBR; 3 Hospital Medicine, Princess Alexandra Hospital, Harlow, GBR; 4 Ophthalmology, Barts and The London School of Medicine and Dentistry, London, GBR; 5 Ophthalmology, Warrington and Halton Teaching Hospitals NHS Foundation Trust, Warrington, GBR; 6 Anaesthetic, Princess Alexandra Hospital, Harlow, GBR

**Keywords:** cognitive inflexibility, dorsomedial striatum, obsessive-compulsive disorder, qpcr, reversal learning, scube1

## Abstract

Background

Obsessive-compulsive disorder (OCD) is associated with reversal-learning deficits and dysregulation within cortico-striatal circuitry. Genetic studies have nominated several OCD risk genes, but their relationship to striatal gene expression and cognitive inflexibility in vivo remains unclear. *Scube1* (signal peptide, CUB, EGF-like domain-containing 1) is a secreted EGF-related glycoprotein linked to vascular/inflammatory signaling; its relevance to DMS mechanisms of behavioral flexibility is unknown. This study aimed to test whether dorsomedial striatum (DMS) expression of candidate OCD genes (with *Scube1* as the primary gene of focus) correlates with perseverative responding during reversal learning in rats.

Methods

Male Lister-hooded rats previously trained on a serial two-hole spatial discrimination with within-session reversals were used. After the final session, DMS tissue was micropunched for SYBR-green, real-time polymerase chain reaction (RT-qPCR). Primers were designed by Primer-BLAST and screened by melt-curve QC; *H3b* served as the reference gene (cyclophilin cross-check). Expression for all targets was 2^−ΔCq^ (H3b-normalised; hemispheres averaged when available). The pre-specified primary endpoint was the correlation between *Scube1* expression (2^−ΔCq^, hemispheres averaged when available) and perseverative errors (average over reversals 1-3). Exploratory correlations were run for *Wdr7, Chd8, *and *Sapap3* (α=0.05).

Results

Primer evaluation yielded four acceptable targets (*Scube1, Wdr7, Chd8, Sapap3*). *Scube1* expression in DMS positively correlated with perseveration (r(39)=0.35; p=0.027). No significant associations were observed for *Wdr7* (r(39)=0.16; p=0.32), *Chd8* (r(39)=0.11, p=0.46), or *Sapap3* (r(39)=0.18; p=0.27).

Conclusions

Higher *Scube1* expression in rat DMS is associated with greater perseverative responding, suggesting a vascular/inflammatory-linked pathway may contribute to cognitive inflexibility relevant to OCD. Findings support using risk-gene-guided targets in animal models to probe OCD pathophysiology. Replication with full qPCR efficiency calibration and prospective power analysis is warranted.

## Introduction

Obsessive-compulsive disorder (OCD) is a neuropsychiatric condition characterised by intrusive thoughts (obsessions) and repetitive behaviours (compulsions) performed to alleviate distress [1]. It affects 1.5-3% of the population and usually begins in adolescence [2]. Family and twin studies estimate heritability at ~40-50%, indicating a substantial genetic contribution [3].

A prominent cognitive account proposes that core symptoms arise, in part, from deficits in cognitive flexibility. Cognitive flexibility refers to the ability to switch between thinking about two different concepts or to switch between different task rules and corresponding behavioural responses. Reversal learning paradigms capture this ability; when previously learned stimulus-response mappings are inverted, impaired flexibility manifests as perseveration. Such deficits are reported across neuropsychiatric disorders and in OCD specifically [4]. In OCD, altered performance on reversal tasks co-occurs with reduced activation within cortico-striatal circuitry, particularly involving orbitofrontal-striatal loops, and can improve with selective serotonin reuptake inhibitor (SSRI) treatment [4-6]. A meta-analysis by Chamberlain et al. [7] of data from 11 OCD set-shifting tasks showed that deficits in cognitive flexibility, especially in extra-dimensional set shifting, were a robust, reproducible finding with a medium-large effect size. Overall, this evidence provides a solid basis for the use of reversal learning tasks as a measure of cognitive inflexibility in OCD.

Converging evidence implicates fronto-striatal substrates in reversal learning. Human imaging and lesion studies highlight orbitofrontal cortex (OFC) involvement [8,9], while the dorsomedial striatum (DMS) also shows recruitment during reversal and lesion-linked perseveration [10]. Together, these findings point to DMS-centred mechanisms as key determinants of behavioural flexibility relevant to OCD.

Genetic studies of OCD, most notably Arnold et al., though historically underpowered at the GWAS level, have nominated several candidates of mechanistic interest [11]. Whole-exome sequencing identified *CDH8* and *SCUBE1* as high-confidence genes in 184 parent-child trios [12]; *CDH8* disruption is associated with repetitive behaviours across species [13,14], while *SCUBE1* is expressed in the developing brain and links to inflammatory biology [15,16]. Most functional work to date has characterised *SCUBE1* in vascular/platelet contexts [15], with experimental evidence for modulation by inflammatory stimuli. Given converging reports of elevated inflammatory markers in youth OCD [16], we therefore prioritised *SCUBE1* a priori as a candidate whose expression in DMS might index a neuroimmune-linked pathway relevant to cognitive inflexibility. Additional evidence implicates *WDR7* [17] and *SAPAP3* (gene: *DLGAP3*; mouse/rat ortholog: *Sapap3/Dlgap3*), a postsynaptic scaffolding protein enriched in striatum; *Sapap3* knockout mice exhibit compulsive phenotypes and corticostriatal synaptic defects, including reversal-learning impairments [18,19]. These observations suggest that gene perturbations affecting corticostriatal signalling, particularly within the DMS, may underlie individual differences in cognitive inflexibility.

However, a key gap remains: most work contrasts diagnostic groups or lesion models and seldom links inter-individual variability in behavioural flexibility to molecular variation within the DMS. Bridging this gene-circuit-behaviour axis in animal models can both test the plausibility of OCD-relevant mechanisms and provide a translational framework for prioritising human risk genes.

This study investigated whether inter-individual variation in reversal learning performance covaries with DMS expression of genes nominated by human genetic studies of OCD (e.g., *CDH8, SCUBE1, WDR7, SAPAP3*). It was hypothesised that poorer reversal learning (greater perseveration) would be associated with altered expression of these candidates within the DMS. To enable transparent replication and future meta-analyses, we report a minimum information for publication of quantitative real-time (MIQE-aligned qRT-PCR) workflow in the DMS, with full primer sequences, quality-control metrics, and per-animal source data provided in the Appendix.

## Materials and methods

The subjects used were the same as those trained by Barlow et al. [20]. RT-qPCR was performed on cDNA synthesised from DMS tissue obtained from these animals. To enable transparent replication and future meta-analyses, a MIQE-aligned RT-qPCR workflow in the DMS is presented, with full primer sequences, quality-control metrics, and per-animal source data provided in the Appendix.

Subjects 

Male Lister-hooded rats (Charles River, UK; 250-300 g at study onset) were housed 4 per cage in temperature- and humidity-controlled rooms on a reversed 12:12 h light/dark cycle (white lights on 19:00-07:00). Water was available ad libitum; during testing, the animals were food-restricted to 85-95% of free-feeding weight. Animals were handled daily for habituation before behavioural training. Allocation to operant chambers and testing order were randomised, and qPCR analyses were conducted blinded to behavioural outcomes where feasible. Inclusion required successful acquisition of the initial spatial discrimination; pre-specified exclusions were one animal that failed acquisition (n = 1) and any ΔCq outliers identified by Grubbs’ test (α = 0.05) in sensitivity analyses. Rats were euthanised post-behaviour. Euthanasia was by CO₂, followed by cervical dislocation. Health and welfare were monitored throughout; no humane endpoints were reached. All procedures were approved by the University of Cambridge AWERB and conducted under UK Home Office project licence 70/8072 in accordance with the Animals (Scientific Procedures) Act 1986. 

Behavioural apparatus

Rats were tested in 12 five-hole operant chambers (Med Associates, Georgia, VT). The testing apparatus was controlled by Whisker Control software (Cardinal and Aitken, 2010). The operant chambers were kept inside fan-ventilated boxes, which assured air renewal and masked background noise - an exteroceptive cue that could influence behaviour in the task. Five-square nose-poke holes were set in the curved wall of each box. An infrared detector was positioned across from each nose poke hole, and a yellow stimulus light was located at the rear of each hole. On the opposite wall, a food magazine was located into which rodent food pellets (TestDiet; Purina, UK) were delivered. The three inner apertures of the chamber were blocked using metal inserts, so only the two outermost holes remained unobstructed.

Behavioural training

Initial habituation involved two 20-minute daily sessions. In each session, the two stimulus lights, house light and magazine light, were illuminated, and the magazine was filled with pellets. After this initial habituation, the rats were trained to nose-poke to trigger illumination of stimulus lights and then respond in the holes for food delivery. This took place in each hole under a fixed-ratio one schedule of reinforcement to a success criterion of 50 correct trials in 20 minutes and then increased to FR2 and FR3 (three nose pokes, one food pellet delivery). This schedule was used to eliminate the possibility of random, accidental nose poke responses. Incorrect responses were unpunished; however, errors resulted in a five-second time-out where all lights were switched off.

Acquisition of spatial discrimination 

After the initial habituation training, subjects were trained on a two-hole spatial discrimination task. A nose poke in the food magazine triggered illumination of both stimulus lights. A sequence of three nose pokes in one of the holes resulted in a reward. Three nose pokes in the 'incorrect' hole resulted in a five-second time-out and no reward. Rats were trained until they achieved a success criterion of nine correct trials across the previous 10 trials. ‘Correct’ and ‘incorrect’ holes were designated randomly and counterbalanced across subjects.

Within-session reversal learning 

This session began with the illumination of both the house light and magazine light. For individual rats, ‘correct’ and ‘incorrect’ holes were those experienced in the initial acquisition of the spatial discrimination described above. After rats had reached the success criterion on this acquisition stage, the contingency was reversed; ‘correct’ and ‘incorrect’ holes were reversed such that the initially rewarded response now resulted in the five-second time-out period, and the previously unrewarded response resulted in the reward of a food pellet. Subjects completed three reversals during the one-hour session. Serial reversal was used because many animals display significant perseveration on the first reversal they undergo. Due to this, a single reversal does not differentiate between animals with good and bad cognitive flexibility and learning ability. Therefore, rats were allowed to complete second and third reversals to obtain suitable measures of perseverative responses.

Sacrifice and RNA extraction 

Subjects were killed humanely by CO_2_-induced asphyxiation and cervical dislocation. Subsequently, brains were removed and cooled on dry ice, with the dorsal brain upmost, before being frozen at -80°C. The brains from the cohort we used for qRT-PCR analysis were flash frozen in isopentane, at -30°C, to ensure minimal RNA degradation and then stored at -80°C. Isopentane was used because it was unlikely to form a vapour upon contacting warm objects and therefore allowed for freezing to be even across the whole brain. Brains were sectioned in the coronal plane into 300-micrometre slices using a Jung CM300 cryostat (Leica, Wetzlar, Germany). Sections were stored at -8°C before being thawed at room temperature for processing. Brain slices were micropunched into 1.2 mm diameter circles. For qRT-PCR analysis, tissue was collected as described and placed in RNA later stabilization reagent (Qiagen, UK) for at least one hour at room temperature before being frozen at -20°C.

Gene expression 

Messenger RNA extraction from DMS was as per the manufacturer’s protocol using the miRNeasy Micro Kit (Qiagen) with additional DNAse digestion. cDNA was generated for the samples from 5 ng total RNA using the RevertAid First Strand cDNA Synthesis Kit (Thermo Scientific, UK). SYBR green-based quantitative RT-PCR was performed on the CFX96 Touch Thermal Cycler (Bio-Rad, UK) as per manufacturer guidelines. Cycling was 95°C for five minutes: 40× (95°C 10 s, 60°C 10 s, 72°C 1 min), followed by a melt-curve 65-95°C. The primer pairs, designed using Primer-BLAST software (NCBI) and purchased from Sigma-Aldrich, are given in Table 2. Normalised relative quantities for all genes of interest were calculated using H3B as a reference gene, which acted as an internal control. The stability of H3B itself was confirmed with a second housekeeping gene (cyclophilin). All quantification curves and melt curves were visualised using Bio-Rad CFX Manager 3.1 (Bio-Rad Laboratories, Hercules, CA). Wells failing melt-curve QC were excluded prior to per-animal averaging; melt pass/fail counts and replicate distributions are summarised in the Appendix in Table 3. 

Primer selection and primer design 

A list of genes of interest (Table 1) was collated using data from a genetic meta-analysis by Smit et al. [17], whole genome sequencing by Cappi et al. [12], and the results of the largest meta-analysis for OCD done by Arnold et al. [11]. Primers were designed for these genes using Primer-Blast software (NCBI), as shown in Table 1. Full forward/reverse sequences, amplicon length, and accession are listed in Appendix Table 2. These were then tested on OFC reference tissues, and the successful primers were those used on the DMS from the reversal learning rat subjects. The threshold for exclusion was a 1/10 cut-off; i.e., in reactions where primer dimers contributed more than 10% of the signal, they were excluded. Specificity was supported by single-peak melt curves and expected amplicon sizes from Primer-BLAST; no post-PCR gel was performed.

**Table 1:** Primers designed for use on reference tissue. *represents successful primer pairs.

Gene of interest	Association with OCD
Sapap3*	Postsynaptic scaffolding protein highly expressed in the striatum is found to be a high risk for OCD in genetic studies, but also used as a knockout mouse model for OCD [18]
Chd8*	Function in several processes that include transcriptional regulation, epigenetic remodeling, promotion of cell proliferation, and regulation of RNA synthesis. Allelic variants of this gene are associated with autism spectrum disorder. Association with OCD found in Cappi et al. [12].
Scube1	Important function in vascular biology, potentially inflammation. Identified as a high-risk gene in Cappi et al. [12]
Wdr7*	Involved in cell cycle progression, signal transduction, apoptosis, and gene regulation. SNPs near Wdr7 loci found to be associated with OCD by Smit et al. [17]
Grik2	This is a glutamate ionotropic receptor. Glutamate signalling is important between the cortex and striatum. Found to be of significance in OCD by Arnold et al. [11]
Slc1a1	Another glutamate transporter implicated in OCD [11]
Dlgap1	Same family as Sapap3 and implicated in OCD by Cappi et al. [12] for its relation to Sapap3, which has been used in knockout models for OCD
Faim2	Anti-apoptotic protein implicated in OCD by Arnold et al. [11] meta-analysis

Data and statistical analysis 

qPCR outputs were Cq values. We defined ΔCq = Cq(target) − Cq(H3B) (H3B = reference). For all target expressions, the expression metric used in analyses and figures was 2^−ΔCq^ (H3B-normalised); when both hemispheres were available, we used the mean of left/right (the exported “Average L/R” value); if only one hemisphere was available, that hemisphere was used.

Behavioural data followed Barlow et al. [20]. Errors were analysed in 10-trial windows. If ≥7 commission errors occurred in a window and this excess was significant by Pearson’s chi-squared (p < 0.05), those errors were labelled perseverative. Perseverative errors were averaged across the three within-session reversals to provide a single index of behavioural flexibility per animal.

The primary analysis tested the association between *Scube1* expression (2^−ΔCq^, Average L/R) and perseverative errors using two-tailed Pearson correlation with 95% confidence intervals (α = 0.05), and no multiplicity adjustment was applied to this pre-specified primary test. Exploratory analyses for other genes used Pearson correlations with Benjamini-Hochberg FDR control across tests (q = 0.05). Although only three exploratory correlations were run, BH was selected a priori to control the expected false discovery rate across these related tests while retaining more power than family-wise procedures (e.g., Bonferroni). To address potential over-conservatism with m = 3, raw p-values are reported alongside BH-adjusted q-values for transparency. Assumptions were assessed by Shapiro-Wilk (normality of variables) and visual inspection of residuals (linearity/homoscedasticity). Grubbs’ test (α = 0.05) was pre-specified for single-point outliers in sensitivity analyses; any exclusions and resulting (n) are reported with the results. Effect sizes are reported as r with 95% CI, p-value, and sample size (n).

Data handling and plots were performed using Microsoft Excel (2017; Microsoft® Corp., Redmond, WA) and GraphPad Prism (2021; GraphPad Software, San Diego, CA). 

qRT-PCR reporting (MIQE summary) 

RNA quality/quantity: A260/280 and A260/230 were checked at extraction; archived per-sample values are not retrievable. Integrity was monitored by single-peak melt curves and replicate consistency.

Replicates and controls: Technical replicates were used; wells failing melt-curve QC were excluded before per-animal averaging, where melt flags were archived. NTC and no-RT controls showed no amplification within the analysed Cq range.

Expression metric (pre-specified for the primary endpoint): For all targets, we used 2^−ΔCq^ normalised to *H3b*; when both hemispheres were available, we used the mean of left/right; if one hemisphere was available, we used that hemisphere. This per-animal value corresponds to the “Average L/R (Gene)” field in the export.

Normalisation: ΔCq = Cq(target) − Cq(*H3b*). *H3b* was cross-checked against tubulin; geometric-mean normalisation yielded similar conclusions.

Primer details and QC: Full primer sequences in Appendix Table 2; melt-QC and replicate distributions in Appendix Table 3; and animal flow and per-gene n in Appendix Table 4.

Data availability: Per-animal ΔCq with behaviour is provided in Appendix Table 5.

Limitations: Primer efficiencies (and R²) were not derived from standard curves for this archived dataset; specificity was supported by single-peak melts and clean NTC/no-RT controls.

## Results

Primer evaluation 

**Figure 1:**
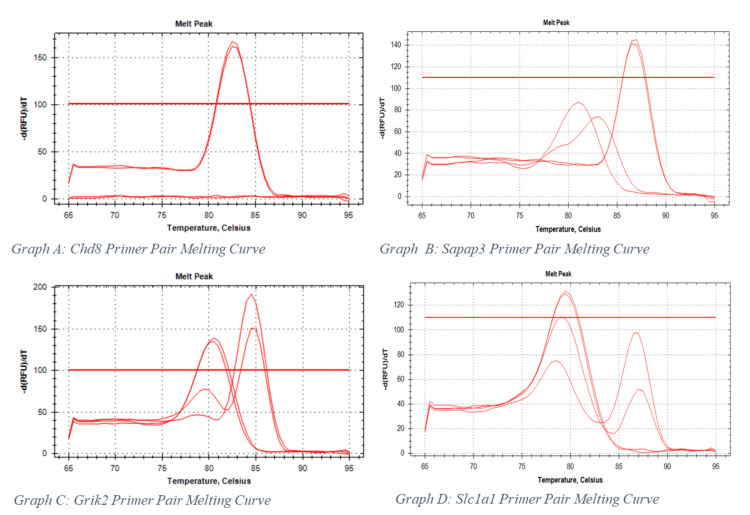
Melting curve analysis for the genes labelled below each graph. There are four signals present in each graph, two are water controls (these present only primer dimer 'noise') and two are the actual reference samples (cDNA from the orbitofrontal cortex of a rat). In each graph, two clearly separable peaks are present; the peak at the lower temperature is due to the presence of primer dimer production, whilst the desired product is the peak at the higher temperature (due to its longer sequence).

Eight primers for genes of interest were tested on the OFC reference tissue (as shown in Table 1). The results were presented as melting curves visualised using Bio-Rad CFX Manager 3.1 (Bio-Rad Laboratories, 2013), as depicted in Figure 1. Graphs A and B (below) depict melting curves of successful primer pairs, with primer dimers producing minimal contribution to the total signal. Primer pairs that produced primer dimers that made up >10% of the total signal were excluded from further testing on the DMS samples; examples of this are shown in graphs C and D (Figure 1). Primer evaluation left four successful primers to be used on the DMS: *Sapap3*, *Wdr7*, *Scube1,* and *Chd8*, as depicted in Table 1. Per-animal values and workflow details are provided in the Appendix (Tables 2-4) with raw per-animal data in Appendix Table 5.

*Scube1* expression in the DMS is associated with elevated perseverative behaviour

**Figure 2:**
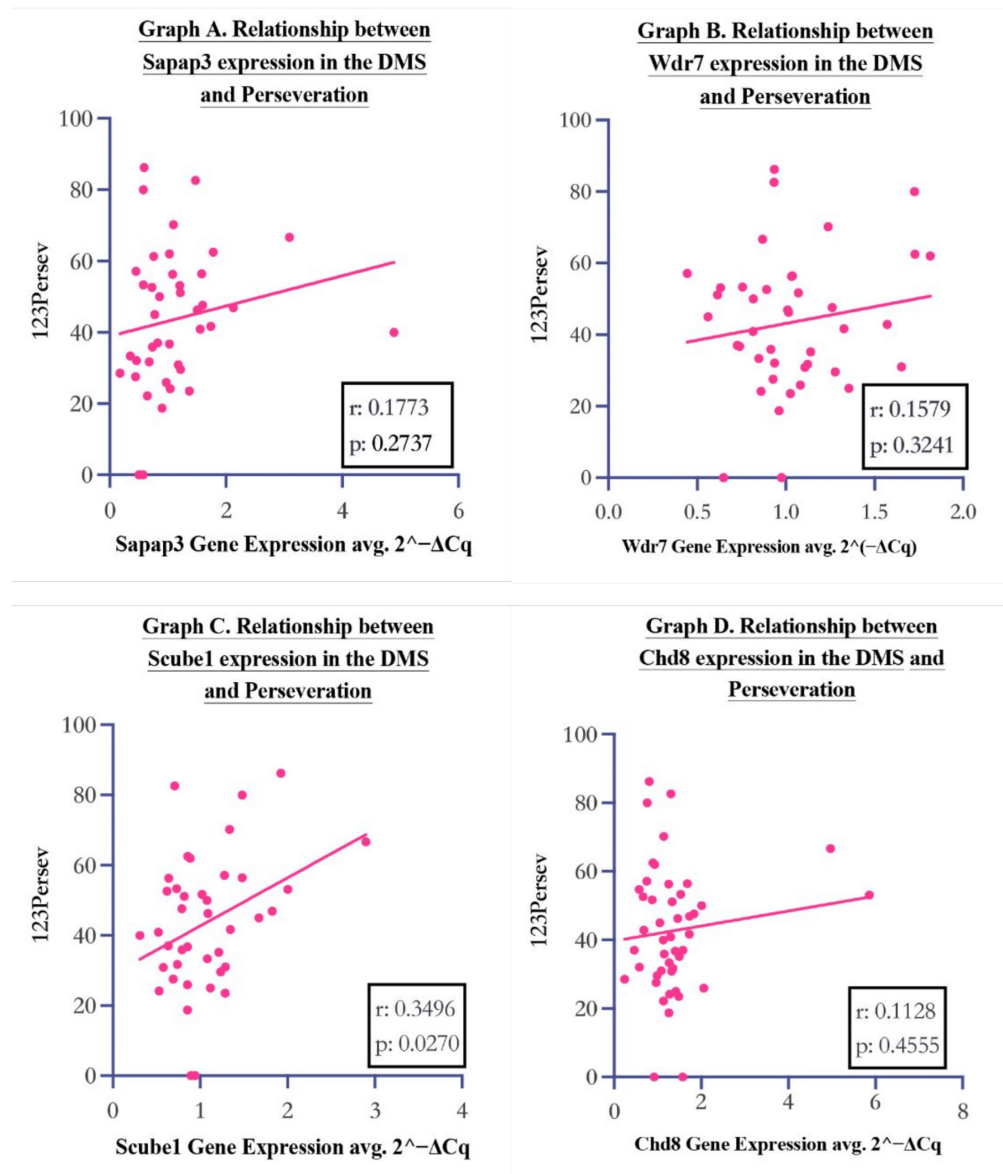
Relationship between DMS gene expression (2^–ΔCq, H3b-normalised) and perseveration. Points show per-animal values (Average L/R where available). Lines are least-squares fits with 95% CI (shaded). Reported are Pearson r, p. Higher 2^–ΔCq indicates higher expression.

There was a significant correlation between *Scube1* expression in the DMS and perseverative behaviour during reversal learning tasks (Figure 2C). This was measured by Pearson’s correlation coefficient (r(39) = 0.35, p = 0.027). 

*Wdr7* expression in the DMS showed no association with perseverative behaviour 

There was no significant correlation between *Wdr7* expression and perseverative behaviour, as confirmed by Pearson’s correlation test (r(39) = 0.16, p = 0.32).

*Chd8* expression in the DMS showed no association with perseverative behaviour 

There was no significant correlation between *Chd8* expression and perseverative behaviour, as confirmed by Pearson’s correlation test (r(39) = 0.11, p = 0.46). This goes against our hypothesis that lower levels of *Chd8* expression in the DMS may be involved with OCD-like behaviour.

*Sapap3* expression in the DMS showed no association with perseverative behaviour

There was no significant correlation between *Sapap3* expression and perseverative behaviour, as confirmed by Pearson’s correlation test (r(39) = 0.18, p = 0.27). Again, this goes against our hypothesis that lower levels of DMS *Sapap3* expression would be associated with increased perseverative behaviour. The implications of these results will be further considered in the discussion.

## Discussion

The main finding of this study provides preliminary evidence that increased *Scube1* expression in the DMS is associated with increased perseverative behaviour. However, inter-individual heterogeneity in the other three genes assessed (*Wdr7*, *Chd8*, *Sapap3*) did not have a significant effect on perseverative behaviour. The results of this study also provide further evidence that animal models of OCD are plausible and provide evidence that identifying risk genes through genome sequencing studies is a pathway to understanding the pathophysiology and underlying causes of OCD, as these same genes are also expressed in animal studies.

A potential role for inflammation in OCD

A significant correlation was found between *Scube1* expression in the DMS and perseverative behaviour of rats in reversal learning tasks. *Scube1* is expressed highly in vascular endothelial cells and is thought to play a role in inflammation [21], and there is preliminary evidence for elevated markers of inflammation found in neuropsychiatric disorders [16]. However, direct evidence for brain endothelial or microglial *Scube1* expression in the adult rat DMS is limited and was not assessed here. More recently, evidence for neuroinflammation in the pathophysiology of OCD is building. There is growing evidence that certain infections are associated with the subsequent onset of OCD symptoms in children, namely, Sydenham’s chorea, pediatric autoimmune neuropsychiatric disorders associated with streptococcal infections (PANDAS), and pediatric acute-onset neuropsychiatric syndrome (PANS) [22]. Sydenham’s chorea has been found to lead to psychiatric symptoms, including obsessive compulsive behaviours [23], which is the reasoning for its link to OCD. The underlying cause of this behaviour is cross-reactivity between antigens in the striatum with group A beta-hemolytic streptococcus (GABHS) antigens, leading to inflammation [24]. However, only a small minority of OCD cases seem to implicate this specific mechanism of infection and subsequent OCD. A second key piece of evidence that inflammation is involved in OCD is the elevated translocator protein (TSPO) levels, which are a marker of gliosis and neuroinflammation, found in the cortico-striatal pathways in patients with OCD [25]. Finally, there is some preliminary evidence that inflammation-modulating medication can be used effectively for OCD [26,27]. However, these are limited in the sense that sample sizes were low, and the impact of the drugs on gliosis should be examined. Accordingly, the present *Scube1* behaviour correlation should be viewed as hypothesis-generating rather than confirmatory. Follow-up work that localises *Scube1* to specific DMS cell types (e.g., brain endothelium, microglia, or neurons) and quantifies protein levels will be required to test an inflammatory mechanism directly. It is important to note that our finding with *Scube1* was suggestive, and further studies into the function of the gene itself are required before coming to a causal link; however, this finding, alongside current literature, has certainly strengthened the argument for neuroinflammation having a role in OCD.

Genetic studies as a guide for further animal studies 

The three remaining genes (*Chd8*, *Wdr7*, *Sapap3*) provided insignificant results and showed no association between levels of expression in the DMS and perseveration in reversal learning tasks. This pattern suggests that baseline DMS mRNA differences for these targets may not map onto reversal-related variability under the present conditions.

Cappi et al. [12] genetic study identified *CHD8* as being a risk gene for OCD, with repetitive behaviours and increased anxiety having also been reported in *Chd8* haploinsufficient mice [14]. One hypothesis from these observations is that *CHD8* may either directly or indirectly alter sensorimotor gating, and rather than reversal learning, *CHD8* may be involved in a different aspect of OCD. Although our study did not find any significant association between reversal learning and expression of *Chd8* in the DMS, further investigation of *Chd8*, with more extensive phenotyping of patients with *CHD8* mutation, would be useful to gain further insight into its potential association with OCD.

*Sapap3* also showed no association with perseverative behaviour in rat reversal learning. *Sapap3* is a postsynaptic scaffolding protein at excitatory synapses that is highly expressed in the striatum. There is significant evidence that links *Sapap3* deficiency with reversal learning impairments. Welch et al. [18] showed that *Sapap3* knockout mice had defects in cortico-striatal synapses, with these same mice showing increased anxiety and grooming behaviour. These findings demonstrate a critical role for *Sapap3* at cortico-striatal synapses and emphasize the importance of cortico-striatal circuitry in OCD-like behaviours. Manning et al. [19] also showed that *Sapap3* knockouts displayed heterogeneous reversal learning, with almost half of the cohort failing to acquire the reversed contingency. Although our results did not support these findings, the *Sapap3* knockout model is useful in emphasising the importance of cortico-striatal circuitry in OCD whilst validating the animal model of perseveration.

The lack of a correlation for *Sapap3* in this study may reflect that small, natural differences in *Sapap3* mRNA in a normal striatum do not produce the large synaptic changes seen when *Sapap3* is completely missing in knockout mice. Moreover, the *Sapap3* function is tightly regulated post-transcriptionally and post-translationally (e.g., isoform usage, synaptic trafficking, phosphorylation/ubiquitination), so bulk DMS mRNA may not track the effective protein abundance at the postsynaptic density where behaviourally relevant signalling occurs. Finally, any relationship might be specific to certain neuron types or synaptic compartments, which can be 'washed out' when measuring whole-tissue homogenates with qPCR. These points together may provide an explanation for the negative result here despite strong effects in *Sapap3* knockouts.

Our findings also provide a framework for more animal studies; identifying risk genes through genetic studies, then testing them against behavioural data, as a way of elucidating more about the underlying pathophysiology of OCD.

Future directions and limitations 

This study aimed to test whether DMS expression of candidate OCD genes correlates with perseverative responding during reversal learning in rats. In doing so, the work supports the validity of this rat model for studying OCD-relevant cognitive inflexibility and helps clarify potential mechanisms underlying perseverative behavior in OCD.

Limited statistical power in existing OCD genome-wide association studies (GWAS) hampers gene discovery. The largest case-control meta-analysis of GWAS to date included ~2,800 cases, which falls well short of the >40,000 cases for many other psychiatric disorders [11]. No significant associations were reported, most likely because the sample size was not big enough for a complex disorder such as OCD. As more research is done in the future on OCD genetics and more risk genes are found, more studies like this can be done to elucidate further the causes of OCD, with this study providing proof of concept that risk genes from genetic studies can be found in animal studies.

Striatal mechanisms have previously been linked to behavioural flexibility through selective lesion studies [28], and the neural pathways are thought to be governed by glutamate signalling. Due to this, glutamatergic gene products may be useful to consider as a potential marker of interest, for example, *Slc1a1*, which has been implicated in OCD. However, although nine different *SLC1A1* polymorphisms have been studied and suggested to be involved in OCD, there are no confirmed susceptibility loci identified thus far [29].

Future work should also quantify *Sapap3* protein in PSD-enriched fractions, assess isoform-specific expression (*DLGAP3* splice variants), and consider cell-type-resolved approaches. Parallel assays in orbitofrontal cortex and corticostriatal terminals, alongside the DMS, may increase sensitivity to circuit-specific effects.

Only male rats were studied. OCD shows sex differences in prevalence, age of onset, and symptom profile, so findings may not generalise to females. Sex hormones and sex-by-gene interactions could modulate dorsomedial striatal gene expression and reversal performance. Future studies should include females, prespecify sex as a biological variable (with sex-stratified or interaction analyses), and power samples accordingly.

Hemispheric data were averaged without formal laterality testing, which may mask left-right DMS differences. Future work should analyse each hemisphere separately to assess potential lateralisation.

Another avenue for further research would be to investigate the fibres running between the OFC and the DMS. Currently, no investigations using fibre sparing lesions have been done, whilst gene expression within these fibres could also be looked at, using retrograde dyes to identify fibres.

## Conclusions

We examined whether genes implicated in OCD by human genetic studies show expression-behaviour relationships in the rat dorsomedial striatum. Among the candidates tested, only *Scube1* expression was significantly associated with perseverative behaviour during reversal learning, whilst *Cdh8*, *Dlgap3* (*Sapap3*), and *Wdr7* were not. This pattern links a vascular/endothelial glycoprotein to an OCD-relevant phenotype and is compatible with emerging roles for inflammatory pathways in compulsivity. However, as the design is correlative and the sample size is modest, these results should be considered preliminary. Future work should replicate in larger, mixed-sex cohorts; include protein-level assays and cell-type-specific localisation; and test causality across corticostriatal nodes. Taken together, our data support the translational relevance of rodent reversal-learning models for probing OCD biology and provide a practical framework: genes prioritised by human studies can be functionally screened against circuit-level behavioural readouts.

## Appendices

All data underlying the conclusions are provided in the supplementary Excel file: Supplements_MCP_Barlow_DMS.xlsx

This workbook contains four labelled sheets:

Sheet 1 content: the exact primer sequences for each gene (Scube1, Wdr7, Cdh8, Sapap3), expected amplicon size (bp), the NCBI RefSeq accession targeted, and whether the amplicon spans an exon junction.

Sheet 2 content: per-well technical replicate Cq values with melt-curve peak temperature and flags (single-peak QC), plus NTC checks. This sheet documents which wells were acceptable for averaging.

Sheet 3 content: sample accounting and final per-gene n, indicating hemisphere availability/inclusion and any exclusion reasons.

Sheet 4 content: the ΔCq values used for analysis, where ΔCq = Cq(target) − Cq(H3B), alongside the behavioural measure (perseverative errors).

**Table 2:** The exact primer sequences for each gene (SCUBE1, WDR7, CDH8, SAPAP3), expected amplicon size (bp), the NCBI RefSeq accession targeted, and whether the amplicon spans an exon junction.

Gene	Primer_Set	Forward_5to3	Reverse_5to3	Amplicon_bp	Accession	Exon_Junction
WDR7	Set1	GCTCTCACCGGACACAAGAG	TGGCTCAGTGTCCTGCAATC	107	NM_001395078.1	Yes (Exon 5–6–7)
WDR7	Set2	ATTATCCGCTCTCACCGGAC	ATTGGCTCAGTGTCCTGCAAT	116	NM_001395078.1	Yes (Exon 5–6–7)
CDH8	Set1	CGGAGCCCGACCTGAGAAA	GAGAGCCGGGAAACAGACAT	92	NM_053393.2	Yes (Exon 1–2)
SCUBE1	Set1	GGACGTGCATCGAGAAGGAT	GTCCTTACACGTCCGGTCAC	149	NM_001134884.1	Yes (Exon 6–7–8)
SCUBE1	Set2	AACTATGGAAACGGAGGCTGTC	TTGCAGCCTCATCCTTCTCGAT	127	NM_001134884.1	Yes (Exon 6–7)
SAPAP3	Set1	GATTCGGACACCGAGAACAGGA	ACGCCAGCCGTCACACTATTGG	113	NM_001276304.1	Yes (Exon 7–8)

**Table 3:** Per-well technical replicate Cq values with melt-curve peak temperature and flags (single-peak QC), plus NTC checks. This sheet documents which wells were acceptable for averaging.

Gene	Melt_OK_Wells	Melt_Fail_Wells	Rats_with_≥1_OK	Median_OK_Replicates_per_Rat	IQR_Q1	IQR_Q3
H3B (reference)	88	6	47	2	2	2
SCUBE1	71	NA	41	2	1	2
WDR7 (Set1)	71	21	41	2	1	2
WDR7 (Set2)	68	23	41	2	1	2
SAPAP3	68	23	43	2	1	2
CDH8	92	2	46	2	2	2

**Table 4:** Sample accounting and final per-gene n, indicating hemisphere availability/inclusion and any exclusion reasons.

Stage	N
Initial cohort	48
Excluded: acquisition failure	1
Available for gene expression (post-acquisition)	47
SCUBE1 ΔCq available	41
WDR7 ΔCq available	45
CDH8 ΔCq available	46
SAPAP3 ΔCq available	43

**Table 5:** ΔCq values used for the analysis, where ΔCq = Cq(target) − Cq(H3B), alongside the behavioural measure (perseverative errors). Note: 2^–ΔCq values are not stored in the file; they were derived from these ΔCq values during analysis.

ratID	Animal_ID	Perseverative_Errors	DeltaCq_SCUBE1	DeltaCq_WDR7	DeltaCq_CDH8	DeltaCq_SAPAP3
1	B420301	45	2.462502608	6.937378689	5.139214864	5.602893946
2	B420302	36.73469388	2.078095287	6.982815478	4.724429622	5.226159233
3	B420303	50	1.922640289	7.132772222	4.228594647	5.458509991
4	B420304	57.14285714	3.048191905	7.57660397	5.981537486	6.582233224
5	B420305	51.11111111	2.366706645	6.936850958	4.780675244	5.056025985
6	B420306	53.33333333	1.877640921	7.302613147	4.709650486	5.619355952
7	B420307	82.60869565	1.812294032	7.159119681	4.843819035	4.905843147
8	B420308	53.125	2.437410131	6.463710037	3.204411669	4.998579056
9	B420309	0	2.276833264	7.596594665	5.36435231	6.208256799
10	B420310	0	1.666581695	7.951984864	4.562768572	6.046590958
11	B420311	31.70731707	1.545548469	7.620555039	4.758654318	5.905184518
12	B420312	24.13793103	1.882289623	7.56567264	4.87172615	5.179343093
13	B420313	61.29032258		6.461187894		6.461187894
14	B420314	37.03703704		6.5702173	6.807610439	6.5702173
15	B420315	28.57142857		7.742556126	7.588221306	7.742556126
16	B420316	54.76190476			6.507728788	
17	B420317	22.22222222		6.264265953	5.247338608	6.264265953
18	B420318	42.85714286	0.216889112		5.760203302	
19	B420319	40.90909091	1.928954919	7.386762371	4.841446857	4.596771418
20	B420320	41.66666667	1.221941408	6.260547582	4.405823513	4.031751338
21	B420321	52.63157895	1.019194863	7.789271803	5.837222946	5.966209126
22	B420322	62.5	0.826420489	6.695441923	5.049258713	4.467093147
23	B420323	86.20689655	1.735604266	7.177977602	5.583085323	6.023951183
24	B420324	56.41025641	1.424021362	6.069802377	6.104160232	4.17343746
25	B420325	46.26865672	1.607912248	6.719727927	4.649541111	4.651313679
26	B420326	35.8974359	1.840972758	7.380543711	4.992985414	5.494825165
27	B420327	29.62962963	1.272693323	6.940346809	5.21206387	5.163170479
28	B420328	33.33333333	1.972179407	7.771480047	5.000663511	6.591939989
29	B420329	30.90909091	1.685889422	7.314350059	5.448855469	4.596230924
30	B420330	80	0.854908783	6.88508566	5.586253457	5.35299966
31	B420331	62	0.873463667	7.176950374	5.504160411	5.265879066
32	B420332	47.61904762	1.296542413	6.878843257	4.315294217	4.131525808
33	B420333	32.0754717	0.574453897	5.487823727	4.591503674	4.822213905
34	B420334	70.1754386	1.323608792	6.783206523	5.004963811	5.086255772
35	B420335	66.66666667	0.443772893	6.393592341	3.388613169	5.206164821
36	B420336	35.18518519	1.442323965	3.714487139	4.777974807	-1.038115156
37	B420337	31.03448276	0.907375382	3.97279159	5.100198092	-0.455981376
38	B420338	56.25	1.695847944	7.375708837	4.980770578	5.187659899
40	B420341	25.92592593	1.70194262	7.498726784	4.992237818	5.439648004
41	B420342	27.5862069	1.516868608	7.224869089	4.213062961	5.297091209
42	B420343	23.52941176	1.825482395	8.218914984	5.249005588	6.976966361
43	B420344	37.03703704	1.593611878	6.665523221	4.616915235	4.793192213
44	B420345	25	2.494925525	7.621493391	4.590320506	5.521123331
45	B420346	51.72413793	1.269453731	8.278314198	4.686070351	
46	B420347	40	1.537466297	8.868835283	5.420019726	
47	B420348	46.875		6.770534879	5.060574591	2.383719941
48			1.658188492	6.169745943	4.39412589	4.29503088
